# Retinal Pigment Epithelial Tear and Retinal Detachment Following Hypotony After Trabeculectomy: A Case Report

**DOI:** 10.7759/cureus.88890

**Published:** 2025-07-28

**Authors:** Yuka Saito, Akira Watanabe, Tomoyuki Watanabe, Tadashi Nakano

**Affiliations:** 1 Ophthalmology, Jikei University School of Medicine, Tokyo, JPN

**Keywords:** choroidal detachment, hypotony, retinal detachment, retinal pigment epithelial tear, trabeculectomy

## Abstract

Hypotony after trabeculectomy (TLE) can lead to choroidal detachment (CD) and maculopathy. We present a case of retinal detachment caused by extensive retinal pigment epithelial (RPE) tears following hypotony following TLE surgery.

A 67-year-old man underwent TLE in the left eye for open-angle glaucoma. Postoperatively, intraocular pressure (IOP) remained stable at approximately 10 mmHg. However, after 50 days, the IOP decreased due to aqueous humor leakage, which required several conjunctival sutures. CD occurred on day 87. On day 108, retinal detachment was detected in the inferior retina of the left eye, and vitrectomy was performed for suspected rhegmatogenous retinal detachment. During surgery, no retinal tears were observed; however, an RPE tear spanning 120° from the 1 o’clock to 5 o’clock position involving the temporal midperiphery to the periphery was observed. The detached RPE was adherent to the underside of the detached retina. Fluid-gas exchange was performed, and subretinal fluid was aspirated from the intentional tears. The next day, IOP was 16 mmHg, and retinal detachment showed improvement. However, by day 5, hypotony recurred due to aqueous leakage, reducing the IOP to 5 mmHg and resulting in redetachment. This required surgical closure of the TLE scleral flap. The following day, IOP increased to 27 mmHg, and the retinal detachment resolved. At the 3-month follow-up, IOP was 16-19 mmHg with medication, and no recurrence of retinal detachment was noted.

Serous retinal detachment may result from RPE tears caused by prolonged hypotony following TLE. Prompt and appropriate management of the IOP may improve retinal detachment and prevent its recurrence.

## Introduction

The advent of a family of procedures collectively referred to as “microinvasive glaucoma surgery (MIGS)” has recently expanded the indications for surgical intervention in eyes with glaucoma [1]. However, certain glaucomatous conditions still require trabeculectomy (TLE) to achieve greater therapeutic efficacy. Severe complications of TLE have been reported, including bleb leaks, choroidal effusions, hypotonic maculopathy, blebitis, and endophthalmitis. Prolonged low intraocular pressure (IOP) owing to hyperfiltration may cause choroidal detachment (CD), retinal choroidal folds, and hypotony [1].

Retinal pigment epithelial (RPE) tears in the macula have been reported following intravitreal injection of anti-vascular endothelial growth factor (anti-VEGF) agents and in conditions such as polypoidal choroidal vasculopathy and retinal angiomatous proliferation [2]. RPE tears in the macula can affect visual function and may be irreversible. However, RPE tears outside the macula are rare. Peripheral RPE tears have been reported as complications of glaucoma drainage surgery, retinal detachment surgery, panretinal photocoagulation, uveal effusion syndrome, panuveitis, trauma, acute retinal necrosis, and both treated and untreated primary and secondary choroidal tumors [2-7]. The mechanism underlying RPE tears is thought to involve mechanical stretching and subsequent rupture of the RPE due to the accumulation of subretinal fluid within the sub-RPE space and elevated hydrostatic pressure within the pigment epithelial detachment (PED) [7].

Recently, a few cases of retinal detachment associated with RPE tears following glaucoma surgery have been reported; however, the detailed pathogenesis and optimal treatment strategies remain unclear [3-7].

Here, we report a case of retinal detachment caused by an extensive RPE tear due to hypotony following TLE.

## Case presentation

A 67-year-old man underwent TLE in the left eye to treat primary open-angle glaucoma. The patient had been receiving three topical medications and one oral medication for bilateral glaucoma management. Preoperatively, the best-corrected visual acuity was 1.2 in the right eye and 0.8 in the left eye. Both eyes were pseudophakic. IOPs were 15 mmHg and 22 mmHg, respectively. Fundus examination revealed large optic disc cupping in both eyes, more pronounced in the left eye, with significant rim thinning. Axial lengths measured 28.28 mm in the right eye and 28.08 mm in the left eye. The central visual field defect in the left eye had gradually worsened over time.

The surgical procedure was uneventful. On postoperative day 4, laser suture lysis was performed, and IOP was maintained at approximately 10 mmHg. The anterior chamber remained deep, and a functioning filtering bleb was observed. By POD 50, the IOP had decreased to 6 mmHg, accompanied by conjunctival leakage and complete flattening of the filtering bleb. Mild choroidal detachment (CD) was also observed. Conjunctival suturing was performed, resulting in bleb reformation, restoration of a deep anterior chamber, and resolution of the CD within two days.

However, by POD 57, although the anterior chamber remained deep, the IOP had further declined to 5 mmHg, with aqueous leakage noted from the limbal conjunctiva. Additional conjunctival and compression sutures were placed; however, the leakage persisted, and the IOP remained at approximately 4 mmHg. On POD 87, a recurrence of CD was documented. Despite further conjunctival suturing, the leakage could not be adequately controlled.

**Figure 1:**
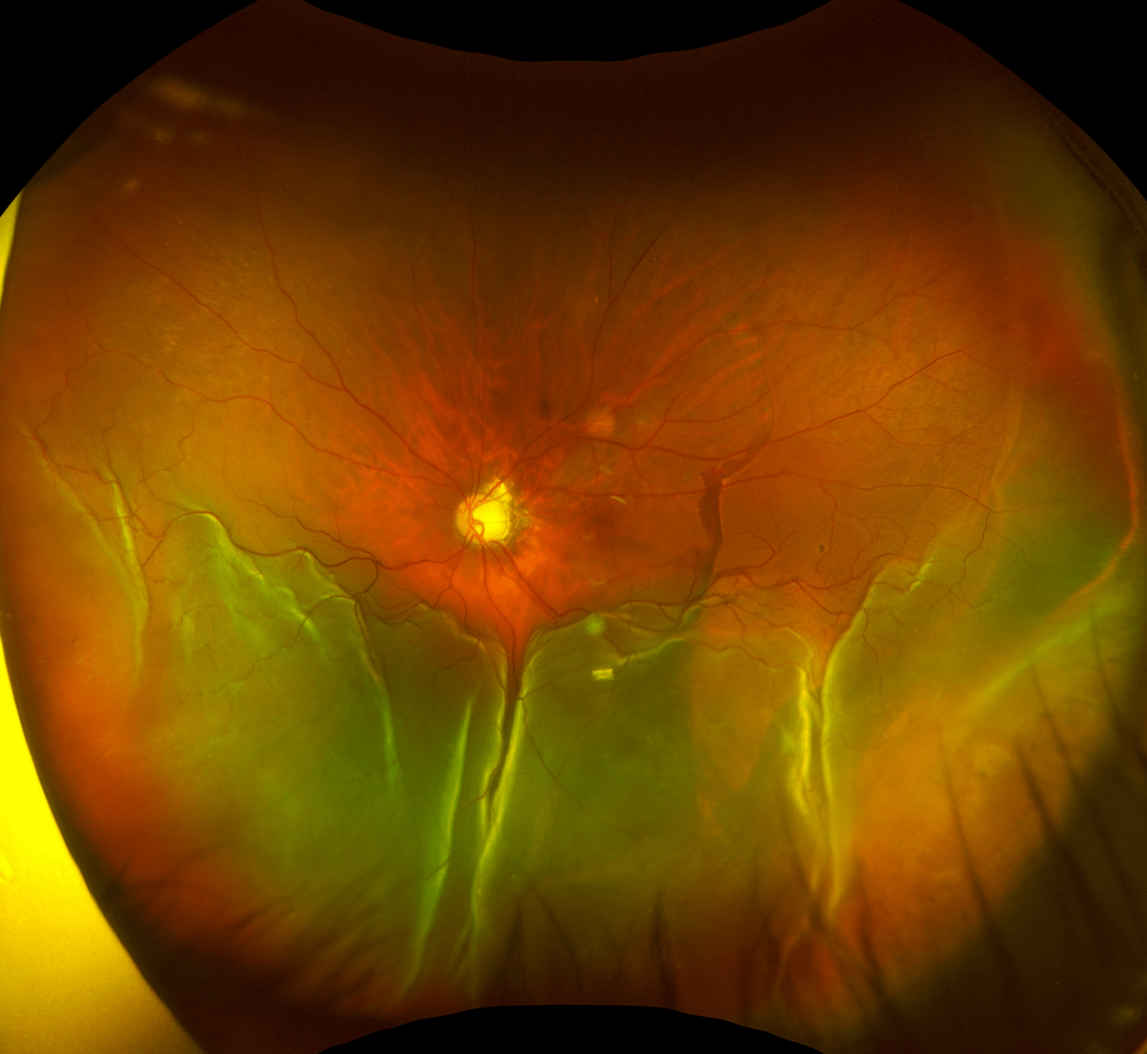
Fundus photograph of the patient’s left eye on POD108 Bullous, macula-off retinal detachment occurs in the inferior retina, overlying a nasal choroidal detachment and a retinal pigment epithelium tear.

Although the glaucoma specialist recommended surgical flap resuturing to manage the bleb leakage, the patient declined further intervention. On POD 108, retinal detachment was observed in the inferior retina of the left eye (Figure 1). Preoperatively, a small inferonasal vitreoretinal tuft was observed without any evidence of retinal tears or breaks, prompting vitrectomy for suspected rhegmatogenous retinal detachment without medical management.

**Figure 2:**
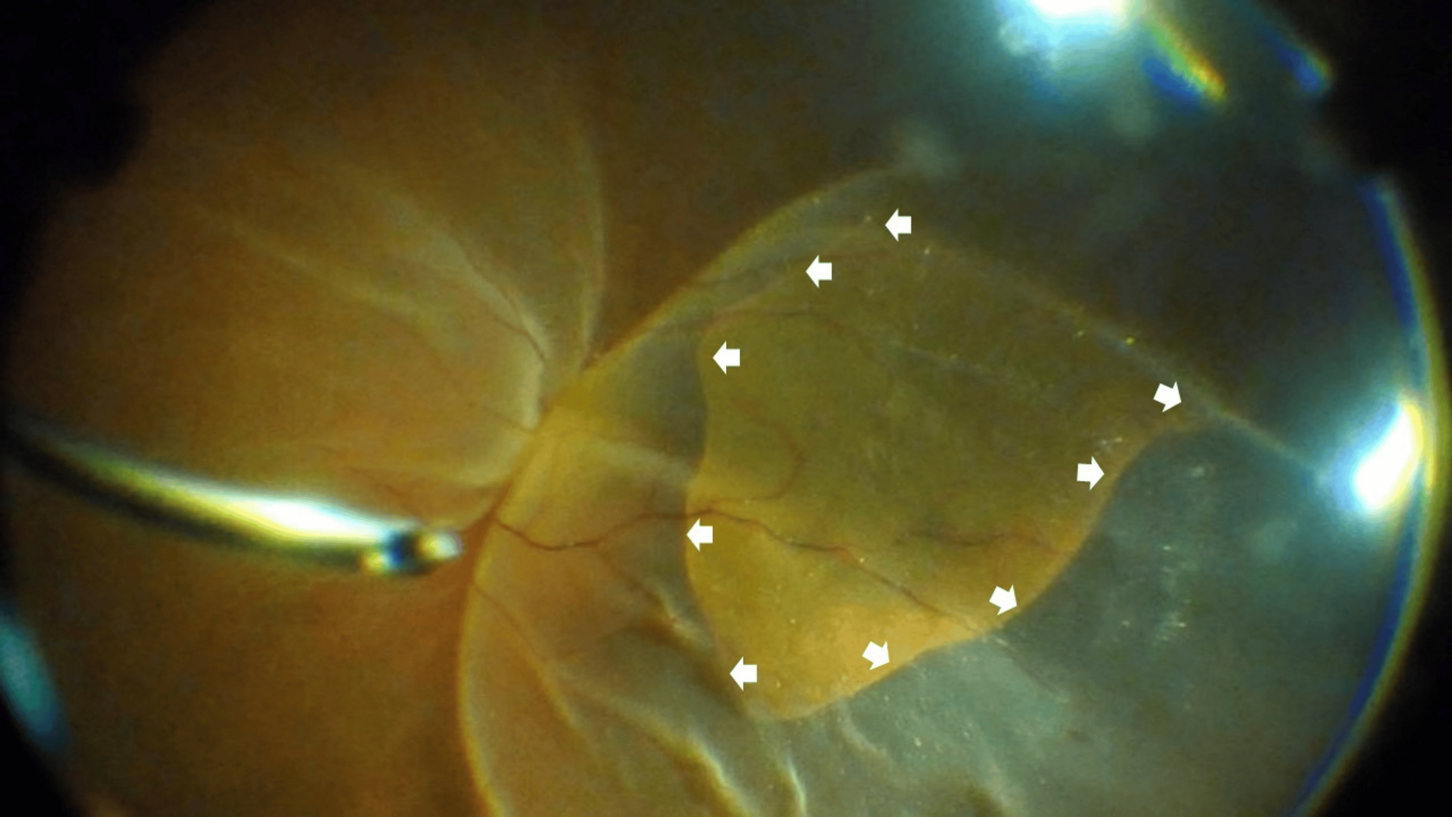
Intraoperative fundus photograph of the patient’s left eye A retinal pigment epithelium tear spanning 120° from the 1 o’clock to the 5 o’clock positions was observed, extending from the temporal midperiphery to the periphery. Arrows indicate a retinal pigment epithelium tear.

Although no retinal tears were observed intraoperatively, an RPE tear spanning 120°, from the 1 o’clock to the 5 o’clock position, was detected from the temporal midperiphery to the peripheral retina (Figure 2).

**Figure 3:**
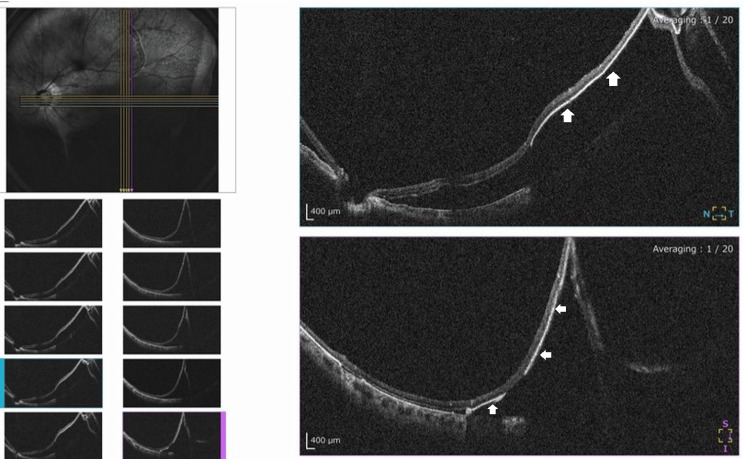
OCT of the patient’s left eye The detached RPE is seen adhering to the underside of the detached retina. Arrows indicate the retinal pigment epithelium. OCT: optical coherence tomography; RPE: retinal pigment epithelial

The detached RPE had adhered to the underside of the retina. No preexisting choroidal neovascularization was identified on optical coherence tomography (OCT) (Figure 3).

A fluid-gas exchange was performed, subretinal fluid was aspirated from the intentional tears, and the vitreous cavity was filled with sulfur hexafluoride (SF6) gas. The next day, IOP was 16 mmHg, and the retinal detachment had improved.

**Figure 4:**
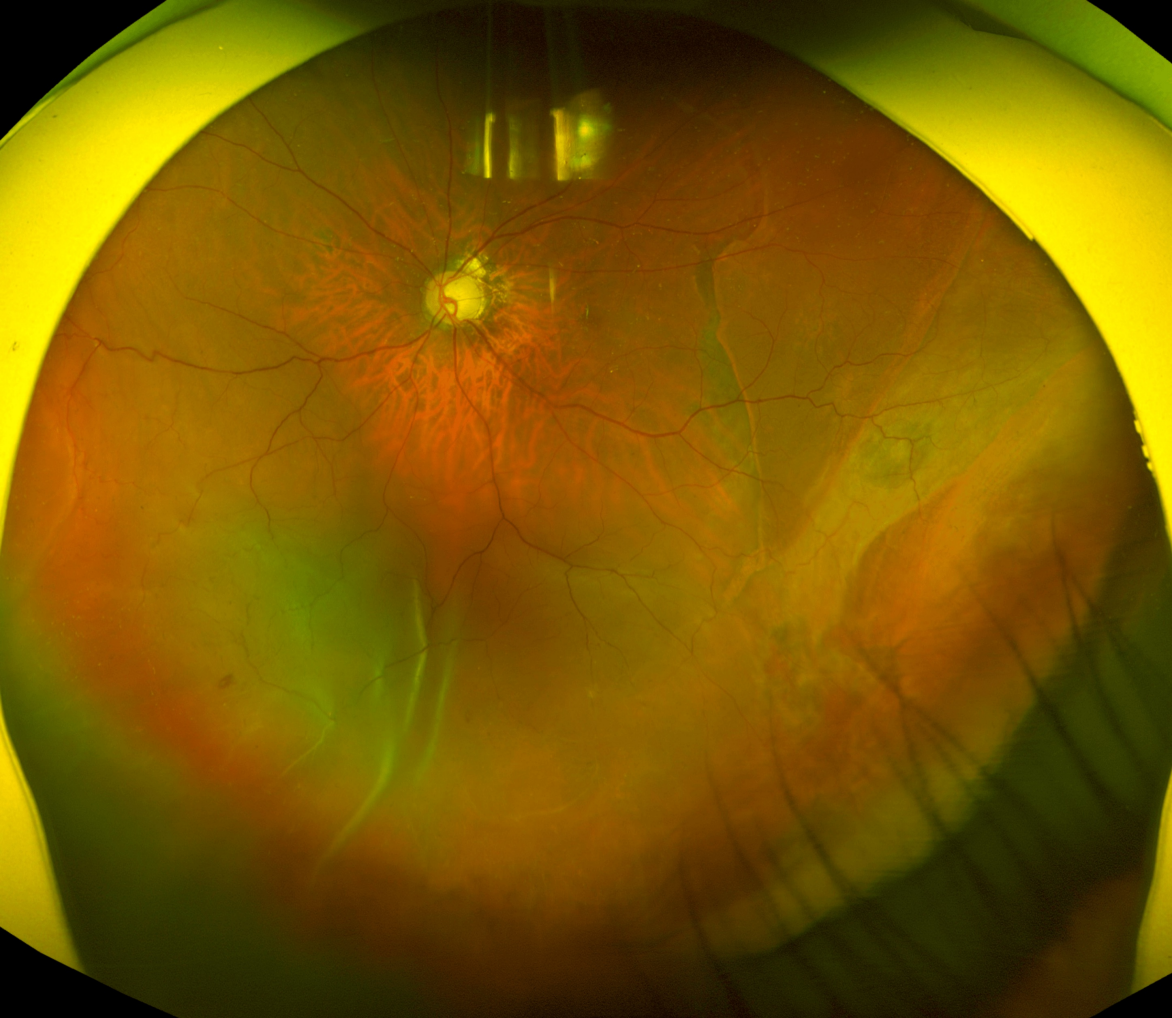
Fundus photograph of the patient’s left eye on POD 21 Recurrent retinal detachment is observed in the inferior retina.

However, on POD 5, hypotony recurred due to aqueous leakage from the TLE bleb, reducing the IOP to 5 mmHg. On POD 8, CD was observed in the temporal inferior retina of the left eye. Although CD persisted on POD 15, no retinal detachment was observed. The patient’s IOP remained consistently low (at 4 mmHg). On POD 21, a recurrence of retinal detachment was observed in the inferior retina of the left eye, accompanied by an IOP of 4 mmHg and a moderately deep anterior chamber. The best-corrected visual acuity in the left eye was 0.02 (Figure 4).

Ocular hypotony was identified as a significant contributing factor to the recurrence of retinal detachment, prompting surgical intervention to close the scleral flap created during trabeculectomy. The conjunctiva was incised, and the leakage site was confirmed. A portion of the scleral flap had melted and was absent, resulting in aqueous humor leakage.

Accordingly, the scleral flap was re-sutured, and the scleral valve was again covered with conjunctiva, which was then sutured in place.

**Figure 5:**
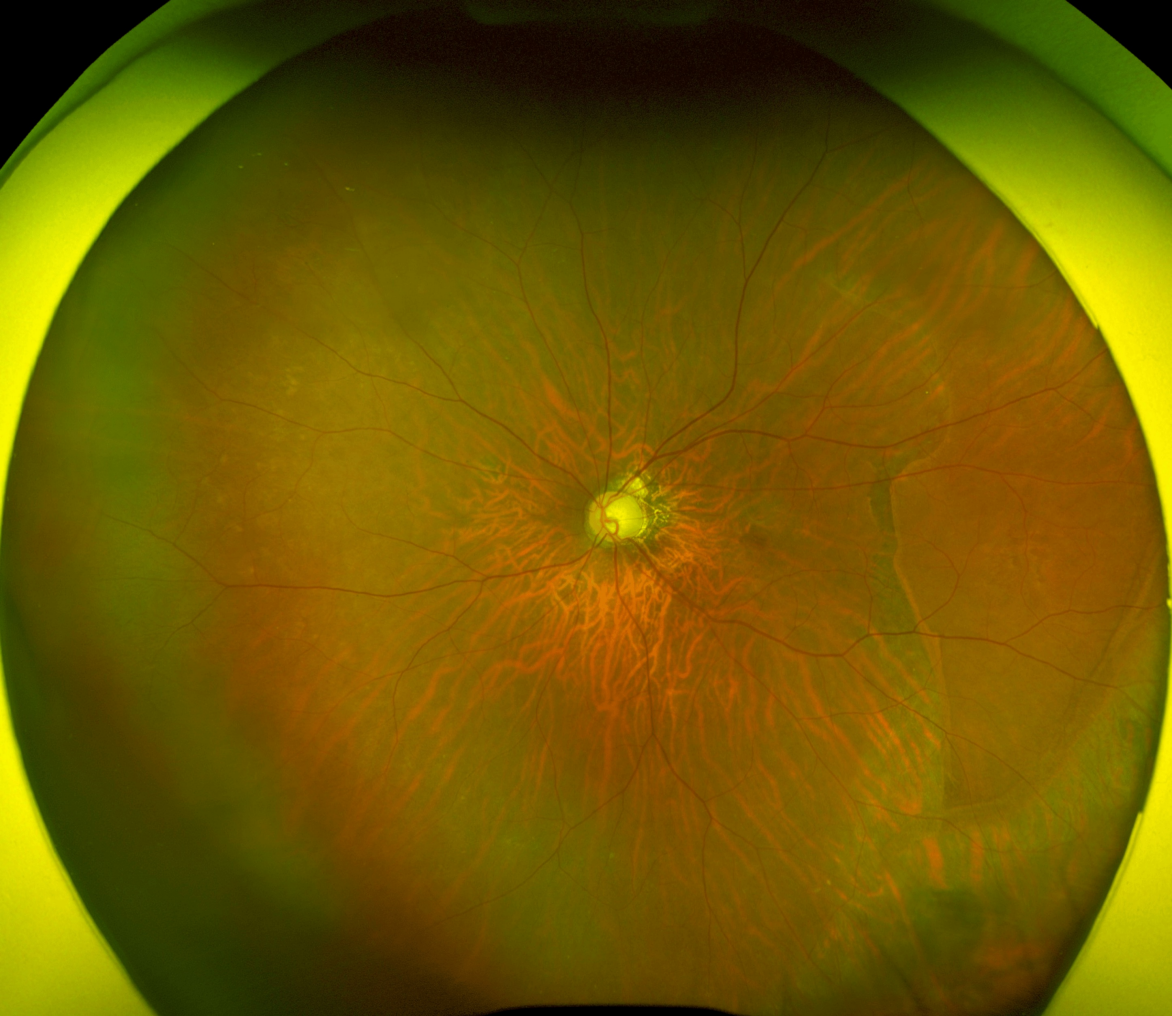
Fundus photograph on the following day

**Figure 6:**
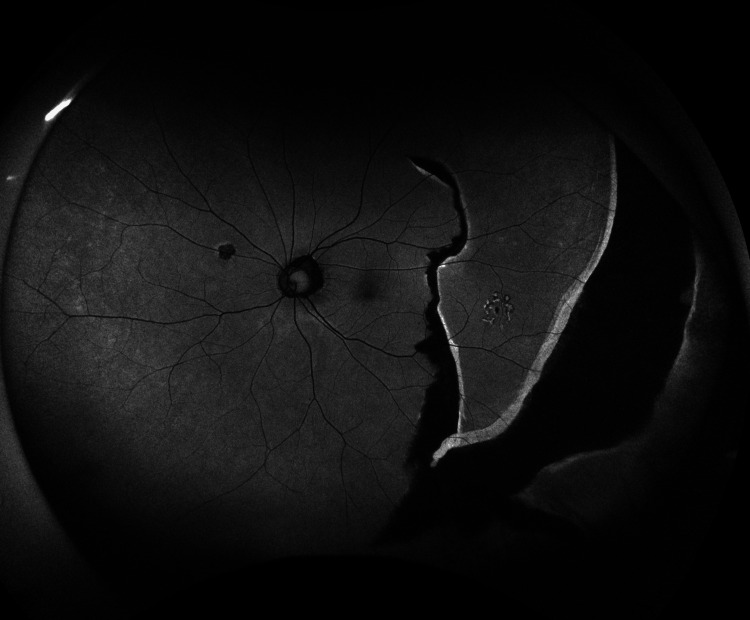
Fundus autofluorescence (FAF) of the patient’s left eye on the following day. FAF reveals a hypofluorescent RPE tear. RPE: retinal pigment epithelial

The day after surgery, except for the V-shaped RPE tear, the other complications resolved without the need for further procedures. On the following day, the IOP increased to 27 mmHg, and the retinal and choroidal detachments were resolved. Fundus autofluorescence imaging revealed the area of RPE tear as hypofluorescent (Figures 5-6). At the 3-month follow-up, IOP was 16-19 mmHg with medication, and no recurrence of retinal detachment was observed. The best-corrected visual acuity was 1.2 in the right eye and 0.5 in the left eye.

## Discussion

RPE tears can develop in the macula following intravitreal injection of anti-VEGF agents for conditions such as age-related macular degeneration, polypoidal choroidal vasculopathy, and retinal angiomatous proliferation [2, 4].

Retinal RPE tears outside the macula are rare. Peripheral RPE tears have been reported as complications of glaucoma drainage surgery, retinal detachment surgery, panretinal photocoagulation, uveal effusion syndrome, panuveitis, trauma, acute retinal necrosis, and both treated and untreated primary and secondary choroidal tumors [4, 5, 7]. These peripheral RPE tears tend to be larger than those in the macula and often exhibit a crescent shape [4].

CD associated with low IOP may play a primary role in the formation of RPE tears. Elevated CD can mechanically stretch the RPE, leading to a rhegmatogenous RPE tear [3, 4]. However, in the present case, there was a discrepancy between the area of significant CD and the location of the RPE tear, suggesting that additional factors may be involved. Notably, CD is not always a prerequisite for RPE tears, as cases have been reported in patients with no history of hypotony [5]. Further investigation into the pathogenesis of RPE rhegmatogenesis is warranted, supported by the accumulation of additional case reports.

Several reports have described serous retinal detachment following RPE tears in cases of low IOP due to hyperfiltration after TLE, similar to the present case [3, 4, 6, 7]. In these reports, CD resulting from prolonged hypotony was believed to permit serous fluid influx through the RPE tears into the subretinal space, causing retinal detachment [3]. Although low IOP may contribute to the development of detachment, RPE rhegmatogenesis remains rare, and the underlying mechanism is not fully understood. While prolonged CD may be associated with the onset of retinal detachment, previous studies have shown variability in the interval between RPE tearing and detachment [3, 4, 6, 7]. In some cases, detachment developed within several days, making it difficult to determine whether persistent CD was the primary cause [6].

Because CD due to low IOP was a major contributing factor in this case, vitrectomy has been reported to be an effective treatment approach [5]. However, the most appropriate management strategy is normalization of IOP. In the present case, although vitrectomy initially restored retinal attachment, the detachment recurred due to persistently low IOP and recurrent choroidal detachment. Ultimately, retinal detachment resolved immediately after the closure of the trabeculectomy flap to prevent excessive filtration, which caused sustained hypotony. In such cases, surgical techniques that aim at preventing low IOP during the initial procedure should be prioritized.

A limitation of this case report is the lack of B-scan ultrasonography, which might have yielded additional insight into the posterior segment status at the time of presentation.

## Conclusions

This case highlights that prolonged hypotony following trabeculectomy can lead to RPE tears and subsequent serous retinal detachment due to fluid migration from the suprachoroidal space into the subretinal space. Retinal detachment resolved immediately after closure of the trabeculectomy flap to prevent excessive filtration, which caused sustained hypotony. Careful management of IOP is essential to promote retinal reattachment and prevent recurrence. Further case studies are required to validate these findings.

## References

[1] Panarelli JF, Moster MR, Garcia-Feijoo J (2024). Ab-Externo MicroShunt versus Trabeculectomy in primary open-angle glaucoma: Two-year results from a randomized, multicenter study. Ophthalmology.

[2] Ersoz MG, Karacorlu M, Arf S, Sayman Muslubas I, Hocaoglu M (2017). Retinal pigment epithelium tears: Classification, pathogenesis, predictors, and management. Surv Ophthalmol.

[3] Harada Y, Okumichi H, Miyata M, Hiyama T, Kiuchi Y (2020). Retinal detachment with retinal pigment epithelial tear under hypotony after trabeculectomy: A case report. Am J Ophthalmol Case Rep.

[4] Laidlaw DA, Poynter R (1998). Giant pigment epithelial tear and exudative retinal detachment complicating choroidal effusions. Am J Ophthalmol.

[5] Rana HS, Cox AR, Biles EC, Broderick K (2022). Giant retinal pigment epithelium tear resulting in neurosensory retinal detachment: An atypical case. J Vitreoretin Dis.

[6] Cutolo CA, Nicolò M, Traverso CE (2022). Retinal pigment epithelium tear after glaucoma surgery. Ophthalmology.

[7] Takemoto M, Kitamura Y, Kakisu M, Shimizu D, Baba T (2023). Retinal pigment epithelial tears after Ex-PRESS filtration surgery in a glaucoma patient with a history of ischemic optic neuropathy. Case Rep Ophthalmol Med.

